# *TMEM126A* mutation in a Moroccan family with autosomal recessive optic atrophy

**Published:** 2012-07-05

**Authors:** Julie Désir, Frauke Coppieters, Nicole Van Regemorter, Elfride De Baere, Marc Abramowicz, Monique Cordonnier

**Affiliations:** 1 Center for Medical Genetics, Hospital Erasme, ULB, Brussels, Belgium; 2 Center for Medical Genetics, Ghent University, Ghent, Belgium; 3 Department of Ophthalmology, Hospital Erasme, ULB, Brussels, Belgium

## Abstract

**Purpose:**

Nonsyndromic autosomal recessive optic atrophy (arOA) is extremely rare and its existence was disputed until a locus, optic atrophy 6 (OPA6), was mapped to 8q. Recently, a second locus, OPA7, was found on 11q in several families from North Africa, with one presumably ancestral mutation of transmembrane protein 126A (*TMEM126A*). Here we report an independently ascertained large consanguineous family of Moroccan descent with three siblings affected with nonsyndromic arOA.

**Methods:**

Assuming autosomal recessive inheritance, we identified a locus on 11q with homozygosity mapping, with a multipoint logarithm of the odds score of 3.84, and sequenced two candidate genes. Direct sequencing of the complete coding sequence of *TMEM126A* revealed mutation p.Arg55X, homozygous in all affected siblings and heterozygous in both unaffected parents.

**Results:**

This mutation was identical to that recently reported in families from North Africa, consistent with a single ancestral origin. In contrast to the recently reported patients, however, the siblings reported in this study had a relatively mild clinical course, with sudden onset in adolescence in the proband. Interestingly, the proband, but not the other affected siblings, had sensory-motor axonal neuropathy with electrophysiological data strongly suggestive of focal demyelinating abnormalities. An unaffected sibling had transient loss of vision after exercise, i.e., Uhthoff's sign of optic neuropathy, and was found to be a heterozygous carrier of the mutation.

**Conclusions:**

Our results confirm genetic heterogeneity in arOA, illustrate clinical variability between families with the p.Arg55X mutation including the description of a mild phenotype in a heterozygote, and underscore the implication of mitochondrial proteins in optic and peripheral neuropathy.

## Introduction

Optic atrophy (OPA) results from degeneration of the retinal ganglion cells whose axons form the optic nerve. Symptoms include a variable association of decreased visual acuity, visual field defects, and color vision abnormalities. The hallmark clinical sign is optic disc pallor. Optic nerve damage is usually irreversible and often progressive. Bilateral and symmetric forms of optic atrophy can be due to nutritional (e.g., vitamin B_12_ or folate deficiency) and toxic insults or to genetic defects [1]. Hereditary optic atrophies can be autosomal dominant, autosomal recessive, X-linked recessive, or maternal (mitochondrial DNA defects). In nonsyndromic optic atrophies, optic nerve degeneration is the only feature of the disease. In syndromic optic atrophies, various neurologic and systemic abnormalities are present. All nonsyndromic optic atrophies characterized to date result from defects in genes encoding mitochondria-related proteins. The most frequent forms of nonsyndromic optic atrophy are autosomal dominant *OPA1*-linked OPA (OPA1, OMIM 165500, prevalence 1/50,000 in several populations [2]) and mitochondrial DNA-linked, maternally inherited Leber hereditary optic neuropathy (LHON, OMIM 535000, prevalence 1/25,000 in northeast England [3]). By contrast, autosomal recessive forms of optic atrophy (arOA) are less frequent, and most cases are syndromic (e.g., OPA3 or Type III 3-methylglutaconic aciduria, Wolfram syndrome, progressive encephalopathy with edema, hypsarrhythmia, and optic atrophy syndrome). Isolated or nonsyndromic arOAs are believed to be extremely rare. They are distinguished from *OPA1*-linked optic neuropathy by the recessive pattern of inheritance, an earlier age of onset (congenital or before age 3), and a generally more severe presentation (possibly including nystagmus or severe dyschromatopsia) [4]. The first locus for isolated arOA, OPA6 (OMIM 258500), has been mapped to chromosome 8q21-q22 in a large multiplex consanguineous family [4]. A second locus was reported on chromosome 11q14.1-q21, and the recurrent nonsense mutation p.Arg55X of transmembrane protein 126A (*TMEM126A*) was shown to be present at homozygous state in the affected patients of four different Maghrebian families [5]. In another Algerian family, affected patients bearing the homozygous p.Arg55X mutation in *TMEM126A* presented with optic atrophy associated with auditory neuropathy [6]. Here we report a novel consanguineous family with arOA and peripheral neuropathy (in the proband only) but without apparent auditory neuropathy, in which we demonstrate linkage to the second reported locus on chromosome 11q14.1-q21, OPA7 (OMIM 612989), and identify the recurrent mutation p.Arg55X at homozygous state in *TMEM126A* in the three affected patients.

## Methods

### Proband and family

**Figure 1:**
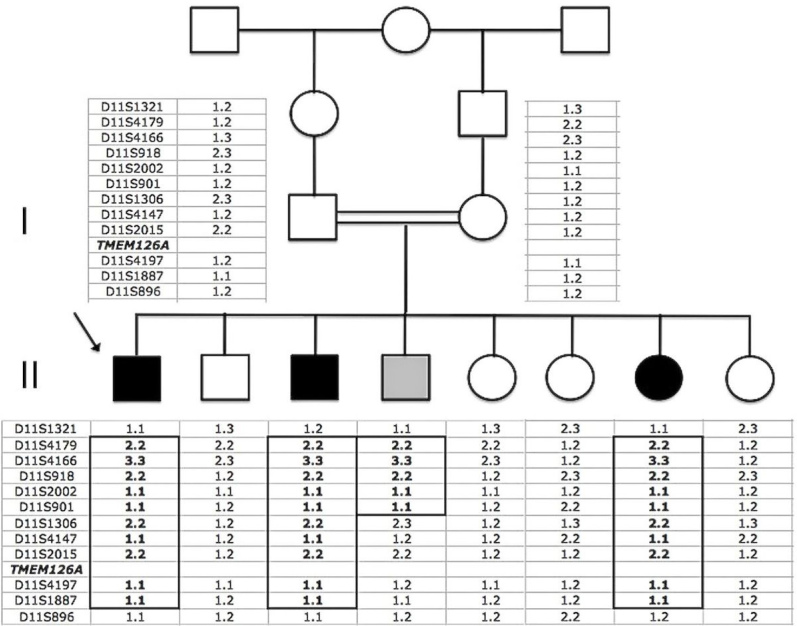
Pedigree of the large consanguineous Moroccan OPA7 family with haplotypes at the 11q13.5-q14.2 region. The parents are half-first cousins with one common ancestor. The arrow indicates the proband. Black symbols represent affected subjects, and the gray symbol represents the brother with Uhthoff’s phenomenon. The homozygosity region is boxed. The distance between D11S1321 and D11S896 is 11.9 cM. The *TMEM126A* position is indicated.

**Figure 2:**
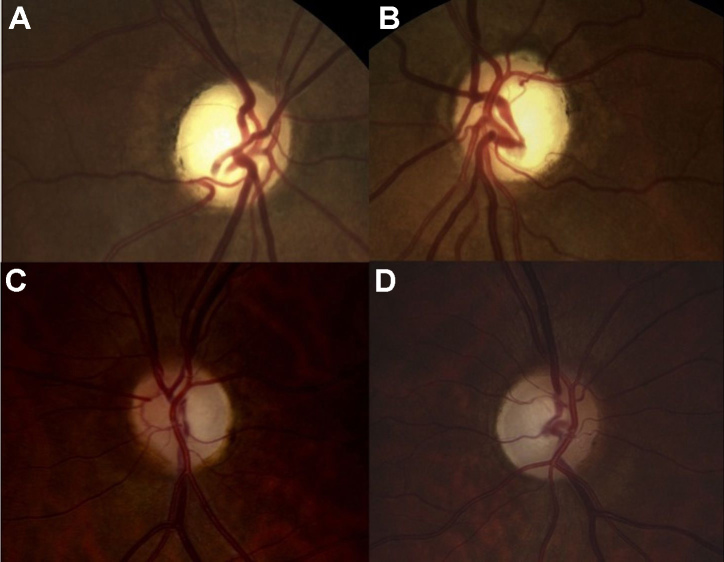
Eye fundi. The right eye (**A**) and left eye (**B**) fundi of II:1 show pale optic discs on both eyes. The right eye (**C**) and left eye (**D**) fundi of II:3 show temporal pallor of both dics.

**Figure 3:**
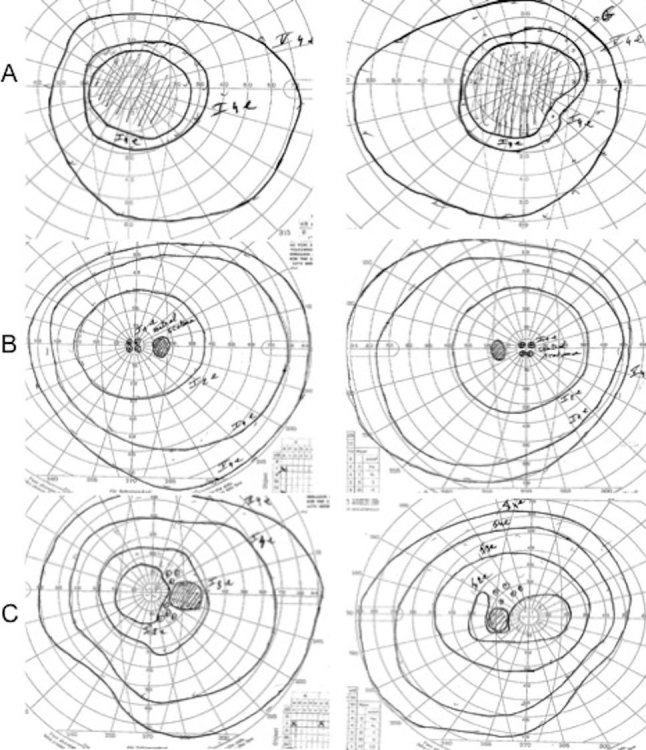
Visual fields. The right and left eye visual fields (Goldmann perimeter are represented for II:1 (**A**), II:3 (**B**), and II:7 (**C**).

**Figure 4:**
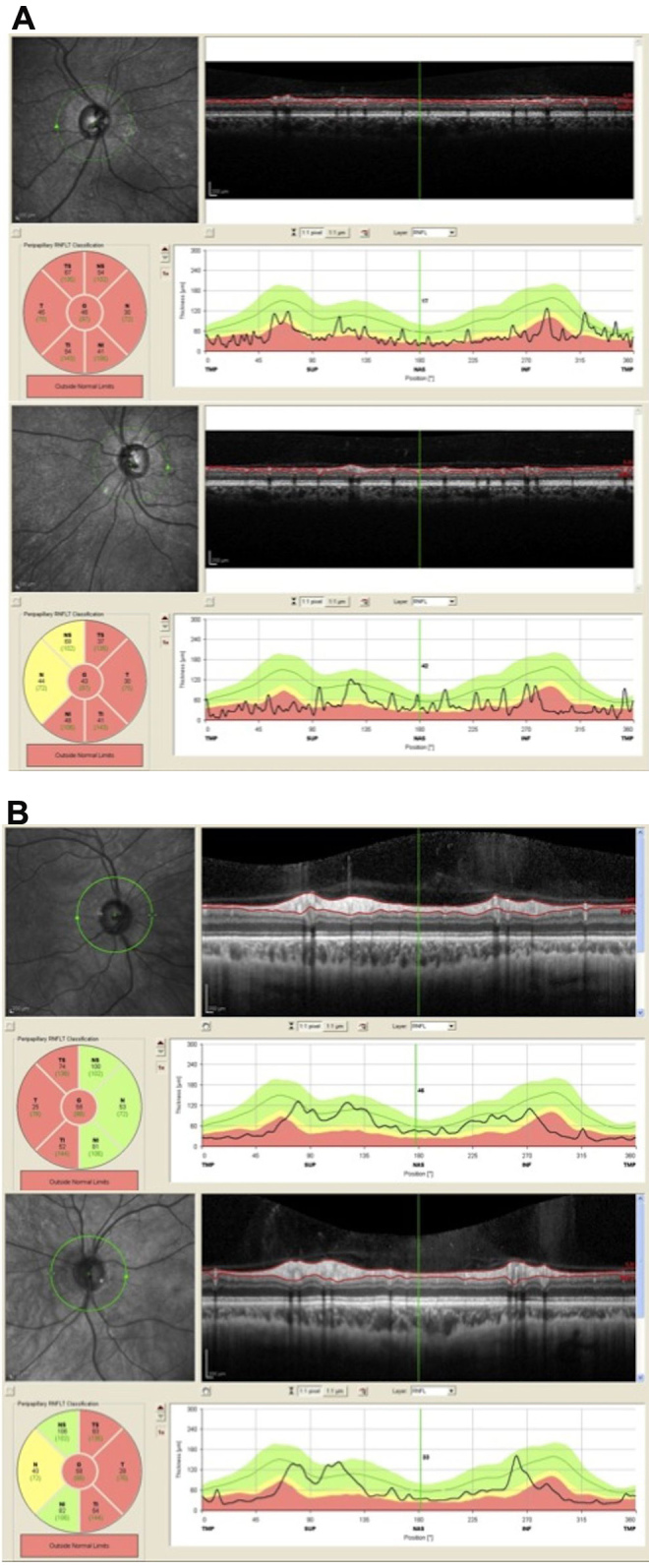
Optical coherence tomography (OCT) of the retinal nerve fiber layers (RNFLs). **A**: The right eye (upper) and left eye (lower) OCT of RNFLs for II:1 show global thinning of all RNFLs. **B**: The right eye (upper) and left eye (lower) OCT of RNFLs for II:3 show temporal thinning of RNFLs in both eyes.

The proband, II:1, a 36-year-old man, was the first child of consanguineous, half-first cousin parents of Moroccan origin (Figure 1). At age 16, he presented with sudden bilateral loss of visual acuity and dyschromatopsia. Leber optic atrophy was suspected because of the brutal loss of vision, although both eyes were simultaneously affected, which is unusual in LHON. No nystagmus was noted. Eye fundus examination, fluorescein angiography, visual field testing, color vision analysis, optical coherence tomography (OCT) scan of the retinal nerve fiber layers (RNFLs), and standard electroretinogram were performed. Table 1 summarizes the clinical findings for the proband. His visual acuity was <20/400 bilaterally (at age 16), the optic discs showed bilateral pallor (Figure 2), the patient had an absolute central scotoma bilaterally (Figure 3A) and severe dyschromatopsia, the standard electroretinogram was normal, and OCT showed thinning of all RNFL bundles (Figure 4A). Fluorescein angiography failed to show the peripapillary microvascular changes that are typical of the acute phase of LHON. Homocysteine and vitamin B_12_ levels were normal in the plasma. The profiles of organic and amino acids in the urine were normal, including a normal level of methylglutaconic acid. The proband had a neurologic evaluation at age 33 years for bilateral weakness of foot dorsiflexion with gait disturbance. Peripheral neuropathy was suspected, and the symptoms improved with intravenous immunoglobulin treatment. The cerebral computed tomography scan was normal. Although the patient had no more complaints, electrophysiological tests were conducted at age 36. An important amplitude reduction was observed on the tibial and sural nerves with conserved conduction velocities and normal F-wave latencies evoking sensory-motor axonal neuropathy. Temporal dispersions were observed on the external popliteal sciatic nerves strongly suggestive of focal demyelinating abnormalities. Two other siblings, one brother, II:3, aged 30, and one sister, II:7, aged 20 (Figure 1), had had visual complaints since primary school, bilateral mild decreased vision, temporal pallor of discs (illustrated in Figure 2, lower, for sibling II:3), relative scotoma in the papillomacular area bilaterally (Figure 3B,C) and dyschromatopsia confirmed with ophthalmological evaluation (see Table 1 for details). The OCT scan of sibling II:3 showed temporal thinning of RNFL bilaterally (Figure 4B). In contrast with the proband, II:1, for which the disease occurred abruptly and the visual loss was severe and did not progress, the disease progressed slowly in the two siblings. The parents and four other children underwent extensive ophthalmological evaluation, and neither signs nor symptoms of optic neuropathy were found. In another brother, II:4, however, mild symptoms were noted, consisting of transient partial visual loss after exercise (Uhthoff’s phenomenon), without pallor of optic discs at funduscopy. Visual acuity was normal (20/15) bilaterally, and color vision tests were normal at age 28 (Table 1). The consanguinity of the asymptomatic parents (half-first cousins with one common ancestor; Figure 1), the absence of symptoms in previous generations and the fact that two males and one female were affected strongly suggested autosomal recessive inheritance.

**Table 1:** Clinical findings.

**Patients**	**Age (years) at onset**	**Age (years) at visual examination**	**VA RE**	**VA LE**	**Fundus**
II.1	16	16	< 20/400	< 20/400	pale optic discs
II.3	primary school	22	20/60	20/60	temporal pallor of discs
II.4		28	20/15	20/15	normal
II.7	primary school	14	20/30	20/25	temporal pallor of discs
	**Visual fields (Goldmann perimetry)**	**ERG**	**VEP**	**OCT**	**Ishihara plates**
II.1	absolute central scotoma both eyes	normal	not done	thinning of all RNFL bundles	none detected
II.3	very small relative central scotoma	not done	not done	temporal thinning of RNFL	20 errors, deuteranopic type
II.4	normal	not done	not done	not done	no error
II.7	short arcuate defects near the blind spot	not done	not done	not done	none detected (but the first)

### DNA extraction

Blood was drawn from peripheral vein with informed consent from all family members. Samples were conserved at room temperature before extraction. DNA was extracted with a standard phenol-chloroform method. All procedures followed the ethical guidelines of our institutions. Our study was approved by the ethical committee of Hôpital Erasme–ULB.

### Mitochondrial DNA analysis

Mitochondrial DNA was amplified to screen for mutations implicated in LHON in the proband and his mother. Mutations of nucleotides 11778 (seen in 40% to 90% of patients with LHON), 3460, 14484, and 15257 (seen in 50% of LHON 11778-negative patients), and of the entire open reading frame of the mitochondrially encoded nicotinamide adenine dinucleotide dehydrogenase 6 gene (MT-*ND6*; seen more rarely in patients with LHON) were screened.

### Linkage mapping

The microsatellites used to exclude the first known locus for autosomal recessive OPA6 on chromosome 8q21-q22 were those described by Barbet et al. [4]. Genomic DNA of the three affected siblings was purified with the QIAamp DNA purification kit (Qiagen, Germantown, MD). DNA was hybridized on a 10K single nucleotide polymorphism (SNP) microarray chip according to the Gene Chip Human 10K array protocol by Affymetrix (Santa Clara, CA). Results were analyzed for large regions of homozygosity [7]. Microsatellite markers were then chosen according to the SNP results for further linkage in all members of the family. Multipoint linkage analysis was performed using the MAPMAKER/HOMOZ algorithm software [8], under the assumption of a fully penetrant disease with an allele frequency of 0.001.

### Candidate gene analysis

Specific primers were designed to amplify and sequence candidate gene segments (primer sequences used to amplify exon 3 of *TMEM126A* were 5′-TGT CAA GAT CGG GAA AGC TC-3′ and 5′-TGC ATT ACA GCA TAC AGC TAC TTG-3′ for a product size of 364 bp). PCR products were purified and sequenced using the Big Dye Terminator cycle sequencing kit v2 (Applied Biosystems, Foster City, CA), and sequencing products were analyzed on a 3100 Genetic Analyzer sequencing machine (Applied Biosystems). The in silico mutation search was performed using the SeqScape software version 2.0 (Applied Biosystems).

## Results

### Mitochondrial DNA analysis

We excluded mutations of nucleotides 11778, 3460, 14484, and 15257 from mitochondrial DNA in the proband and his mother. Mutations of the MT-*ND6* gene were also excluded in the second brother affected, making LHON unlikely in this family.

### Linkage mapping

We first excluded homozygosity for the first known locus for autosomal recessive OPA6 on chromosome 8q21-q22 [4] in the three affected subjects. We did a genome-wide search for homozygosity-by-descent using a 10K SNP array chip. In II:1, we found nine regions larger than 5 Mb, three of which contained more than 500 SNPs (Table 2). After the three affected subjects were compared, only one large region of homozygosity of 10.8 Mb was concordant, on chromosome 11 between rs2226615 and rs2048973 (11q13.5–11q14.2). Further analysis of this chromosomal segment in all members of the family using microsatellite markers confirmed homozygosity in the three affected subjects, and showed homozygosity for a portion of this locus in brother II:4 with temporary partial visual loss following exercise, making it difficult to determine his clinical status (Figure 1 in gray). We did not find homozygosity in the parents or in the unaffected siblings (Figure 1). Multipoint linkage analysis with the MAPMAKER/HOMOZ algorithm software provided a maximum multipoint logarithm of the odds score of 3.84.

**Table 2:** Homozygous regions of more than 5 Mb in II.1

**Homozygous regions**	**Chromosome**	**Length in Mb**	**# SNPS**	**SNP start**	**Position start**	**SNP stop**	**Position stop**
1	9	30	14	SNP_A-2078117	38736473	SNP_A-1812761	68741861
**2**	**11**	**27.44**	**3374**	**SNP_A-4221532**	**76029213**	**SNP_A-2155922**	**103473380**
3	1	23.36	20	SNP_A-2198638	1.2E+08	SNP_A-4238570	143576984
4	16	14.72	84	SNP_A-2303925	31767900	SNP_A-1835934	46484390
5	3	10.63	804	SNP_A-1831302	1.79E+08	SNP_A-1837638	189682173
6	2	9.71	979	SNP_A-2125652	2.18E+08	SNP_A-2021900	2.27785006
7	3	5.84	50	SNP_A-2143755	89819382	SNP_A-1841424	95661425
8	2	5.85	11	SNP_A-1932841	89750715	SNP_A-2108592	95603500
9	5	5.44	71	SNP_A-2213328	44662567	SNP_A-4210939	50098368

In the region indicated in **bold**, the *TMEM126A* gene was found

### Candidate gene analysis

**Figure 5:**
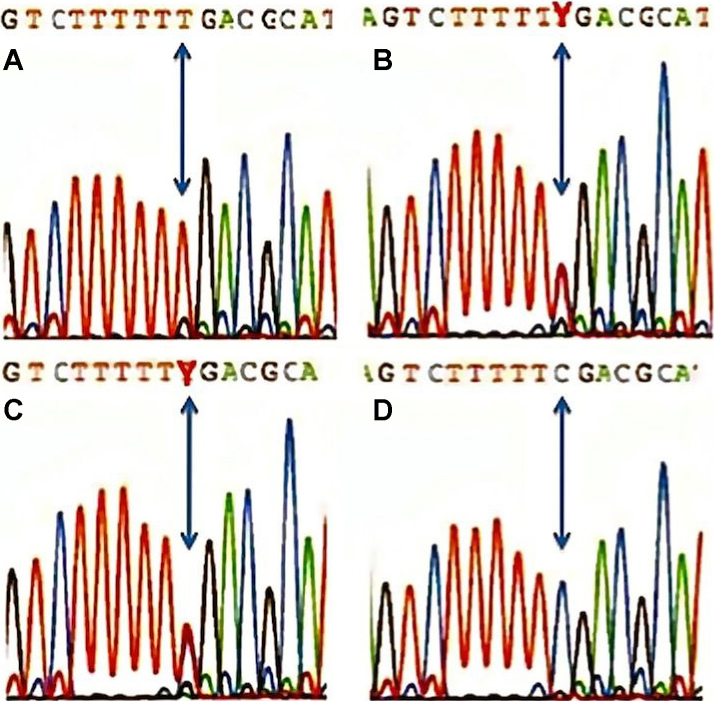
*TMEM126A* sequence profiles. **A**: The proband is homozygote for the mutation c.163C>T (p.Arg55X). **B**: The father is heterozygote for the mutation. **C**: The brother presenting transient partial visual loss following exercise (Uhthoff’s phenomenon) is heterozygote for the mutation. **D**: This unrelated control subject has no mutation.

Forty-six genes were known or annotated in the interval. A nuclear gene encoding a mitochondrial protein, *NDUFC2* (nicotinamide adenine dinucleotide: ubiquinone oxidoreductase [complex I], the first enzyme complex in the electron transport chain of mitochondria), was considered an interesting candidate. We analyzed this gene with direct sequencing using intronic primers flanking each exon. No mutation was found. *TMEM126A* was located in the interval, and a mutation of this gene was reported in autosomal recessive optic atrophy [5] during the course of our study. We sequenced *TMEM126A* in our patients and found the same mutation, c.163C>T (p.Arg55X; NM_032273), as previously reported in four families from the Maghreb [5,6]. This mutation was homozygous in the affected siblings (Figure 5A, proband), heterozygous in the parents (Figure 5B, father), as well as in the brother presenting temporary partial visual loss following exercise (Figure 5C). This nonsense mutation was not found in 100 controls of the same ethnic group (Figure 5D). We genotyped 12 microsatellite markers surrounding TMEM126A in the proband and compared the allele sizes with the affected individuals described by Hanein et al. [5] and Meyer et al. [6]. Interestingly, the two most closely linked microsatellite markers, D11S1354 and D1SS1887, showed only a small difference in allele size with the haplotype reported by Hanein et al. [5], possibly due to inter-run differences (Table 3).

**Table 3:** Alleles sizes of microsatellite markers compared to Meyer et al. [6] and Hanein et al. [5].

** **	**Proband, II.1**		**[**6**]**	**[**5**]**			
**Microsatellite**	**size**	**size-M13**	**Size**	**F1**	**F2**	**F3**	**F4**
D11S937	253,06	**234,06**	163 165	**nd**	**nd**	**nd**	**nd**
D11S918 (AFM203vg1)	210,6	**191,6**	**nd**	183 191 197	**nd**	**nd**	**nd**
D11S4143 (AFMb055yd1)	226,16	**207,16**	**nd**	209 219	207	209	211
D11S1362 (AFMa132xh9)	219,35	**200,35**	**nd**	197	201	201	197
D11S2002	266,19	**247,19**	239	**nd**	**nd**	**nd**	**nd**
D11S1396	187,06	**168,06**	152	**nd**	**nd**	**nd**	**nd**
D11S901 (AFM063yg1)	192,93	**173,93**	310	160	168	176	160
D11S1354 (AFM338xe1)	193,94	**174,94**	**nd**	**177**	**177**	**177**	**177**
**TMEM126A**							
D11S1887 (AFMa049wa5)	278,91	**259,91**	**nd**	**263**	**263**	**263**	**263**
D11S1780 (AFMa082wb9)	189,58	**170,58**	**nd**	189	191	173	173
D11S4176 (AFMb354xa5)	264,81	**245,81**	**nd**	230 214 224	**nd**	**nd**	**nd**
D11S4108	128,26	**109,26**	126	**nd**	**nd**	**nd**	**nd**

Overview of allele sizes of 12 microsatellites genotyped in the proband of this study and the affected individuals described in Meyer et al. [6] and Hanein et al. [5]. The size-M13 represents the allele size minus the length of the M13 tail used (CACGACGTTGTAAAACGAC). In bold underlined: founder haplotype described by Hanein et al. [5]. Abbreviations used: nd: no data; F: family.

## Discussion

The clinical picture of these three siblings showing optic neuropathy involving the papillomacular bundles bilaterally favors the diagnosis of a hereditary optic neuropathy.

The existence of isolated recessive OPA has long been discussed, but in 2003 the first family with an unambiguous autosomal recessive form was described in a large consanguineous family of French origin, linked to a chromosomal locus on 8q [4]. Our data confirm genetic heterogeneity within arOAs: we report a large Moroccan family with recessive OPA not linked to the 8q locus. Linkage analysis in the large family reported here allowed us to delineate a locus on chromosome 11q13.5-q14. All genes involved in hereditary optic neuropathies known to date are mitochondrial (LHON) or nuclear genes encoding proteins with mitochondrial targeting (OPA1, OPA3) [9]. For this reason, we initially screened the *NDUFC2* gene but found no mutation. Our linkage interval encompassed *TMEM126A*, recently implicated in OPA7 [5]. *TMEM126A* encodes a mitochondrial protein of higher eukaryotes with four transmembrane domains and a central domain conserved with TMEM126B [5]. Reverse transcriptase–PCR on total RNA from various adult and fetal human tissues showed that TMEM126A is strongly expressed in the brain (whole), cerebellum, fetal brain, skeletal muscle, testis, fetal retinal pigmentary epithelium, and fetal retina [5]. In situ hybridization to the adult mouse retina at 8 months of age detected significant levels of specific mRNA in the ganglion cell layer, the optic nerve head, the outer plexiform layer, and in the outer ellipsoid length of photoreceptor inner segments [5]. Faint to no labeling was noted in the outer nuclear layer and photoreceptor outer segments [5]. We confirmed the implication of *TMEM126A* in the disease, showing the same biallelic p.Arg55X mutation as the one recently described in the four arOA families from the Maghreb [5]. In addition, as the same mutation was recently identified in two siblings with arOA and auditory neuropathy, originating from a consanguineous Maghreb family [6], which prompted the authors to postulate that *TMEM126A* could also be expressed in inner hair cells. Our patients have no hearing complaints but were not tested for auditory neuropathy. We genotyped closely linked markers, assuming a founder mutation. Although we could not obtain DNA samples from the previously reported patients for comparison in the same analysis, our results are possibly consistent with an ancestral Maghreb mutation and either short tandem repeat increment mutation or differences in calibration. Our study sustains the importance of this gene in arOA. In addition, *TMEM126A* might be an important candidate gene to screen in patients with isolated nonsyndromic optic atrophy, especially in juvenile forms and in patients of Maghrebian origin.

Unlike the patients previously reported with *TMEM126A*-associated OPA7 [5,6], who presented with onset during childhood (between age 4 and 6, and from birth, respectively), and with a severe phenotype, the patients described here presented with a later onset and milder form without apparent cardiac symptoms or hearing defect [6]. Furthermore, the proband in the present family presented with an abrupt onset of symptoms initially thought to be consistent with LHON. His two affected siblings had mild visual problems in childhood but reported slowly progressing loss of vision. Our patients were not tested for hypertrophic cardiomyopathy, minor brain magnetic resonance imaging alterations, or mild hearing loss [5], so these clinical features may have been missed.

We noted sensory-motor axonal neuropathy with electrophysiological data strongly suggestive of focal demyelinating abnormalities in the proband. This phenotypic association of peripheral neuropathy with optic atrophy is also present in hereditary motor and sensory neuropathy type VI with optic atrophy (OMIM 601152) caused by mutations in mitofuscin 2 (*MFN2*) [10], encoding another mitochondrial protein, emphasizing the important role of mitochondrial function for optic atrophies and peripheral neuropathies. Hanein et al. [5] overexpressed a TMEM126A-myc fusion protein into COS-7 cells. Epitope-tagged wild-type TMEM126A colocalized with mitochondrial complex II subunit 70 kDa Fp (SDHA), complex IV subunit 1 (MTCO1), ATP synthase subunit beta (ATP5B), and ATP synthase subunit alpha (ATP5A), supporting the mitochondrial localization of the protein. TMEM126A was suggested to be a mitochondria-localized mRNA (MLR) protein and may be essential in the early nucleation process of large mitochondrial complexes [5]. Interestingly, patients affected with inborn defects caused by mutations in the nuclear genes ATP synthase mitochondrial F1 complex assembly factor 2 (*ATPAF2*), transmembrane protein 70 (*TMEM70*), or ATP synthase, H^+^ transporting, mitochondrial F1 complex, epsilon subunit (*ATP5E*) or in the mitochondrial genes ATP synthase 6, mitochondrial (*MTATP6*) or ATP synthase 8, mitochondrial (*MTATP8*), encoding proteins of the mitochondrial complex V (ATP synthase), whose subunits have also been shown to be MLR proteins [11], present with neonatal-onset hypotonia, lactic acidosis, hyperammonemia, hypertrophic cardiomyopathy, 3-methylglutaconic aciduria, and in some cases peripheral neuropathy [12].

We report the first observation of a heterozygous carrier of the p.Arg55X mutation complaining of partial vision loss following exercise (Uhthoff’s phenomenon), but displaying no pallor of optic discs at funduscopy. Uhthoff's phenomenon is typically associated with optic neuritis in multiple sclerosis [13]. The relatively mild clinical course in our patients is similar to the 8q-linked OPA6 reported in a French family [4], but our patients had severe dyschromatopsia and bilateral central scotoma. We conclude that the p.Arg55X mutation in *TMEM126A* is probably ancestral in North African populations. Homozygotes may present with an abrupt onset of symptoms that mimic LHON or the course may be mild with childhood or adolescent onset. Finally, Uhthoff’s phenomenon might be a clue to the heterozygosity of the mutation in unaffected family members.
